# Jasmonic Acid Effect on *Cucumis sativus* L. Growth Is Related to Inhibition of Plasma Membrane Proton Pump and the Uptake and Assimilation of Nitrates

**DOI:** 10.3390/cells12182263

**Published:** 2023-09-13

**Authors:** Małgorzata Janicka, Małgorzata Reda, Emilia Mroczko, Anna Wdowikowska, Katarzyna Kabała

**Affiliations:** Department of Plant Molecular Physiology, Faculty of Biological Sciences, University of Wrocław, Kanonia 6/8, 50-328 Wrocław, Poland; malgorzata.janicka@uwr.edu.pl (M.J.); malgorzata.reda@uwr.edu.pl (M.R.); 308927@uwr.edu.pl (E.M.); anna.wdowikowska@uwr.edu.pl (A.W.)

**Keywords:** environmental stress, growth inhibition, hydrogen peroxide, jasmonic acid, NAR protein, nitrate transporters, nitrate reductase, plasma membrane H^+^-ATPase, proton pump, ROS

## Abstract

When plants are exposed to environmental stress, their growth is inhibited. Under such conditions, controlled inhibition of growth is beneficial for plant survival. Jasmonic acid (JA) is a well-known phytohormone that limits plant growth, which has been confirmed in several species. However, its role in cucumber seedlings has not yet been comprehensively investigated. For this reason, we aimed to determine the involvement of JA in the regulation of proteins crucial for growth including plasma membrane proton pump (PM H^+^-ATPase), PM nitrate transporters, and nitrate reductase (NR). Treatment of cucumber seedlings with JA not only limited their growth but also increased the H_2_O_2_ content in their roots. The main sources of ROS generated for signalling purposes are PM NADPH oxidase (RBOH) and superoxide dismutase (SOD). Exposure of seedlings to JA induced the expression of some *CsRBOH* and SOD encoding genes, suggesting that ROS signalling can be activated by JA. As a consequence of JA exposure, the activity of all analysed proteins was inhibited and the expression of their genes was modified. The results indicate that reduction of PM H^+^-ATPase activity and the related decrease in nitrate uptake and assimilation are responsible for the root growth retardation of JA-treated plants.

## 1. Introduction

Plants are constantly exposed to unfavourable environmental conditions. It is very important to analyse in detail how plants deal with the balance between growth and defence [1]. In developmental physiology, growth is understood as a process of irreversible enlargement of a plant body. It occurs in specific parts of the plant due to cell division and increased volume. Plant growth is a complex process in which plasma membrane proton pump (PM H^+^-ATPase) plays an important role. PM H^+^-ATPase belongs to subfamily III of P-type ATPases present in the cell membranes of plants and fungi [2]. Both the N- and C-termini of the protein are located on the cytoplasmic side [2,3]. Although plasma membrane proton pump is present in all types of plant cells, the expression levels of its specific isoforms vary depending on the species, stage of development, and organ [4]. Under physiological conditions, the main function of PM H^+^-ATPase is to generate the energy necessary for secondary transport. The resulting electrochemical gradient maintains a potential difference across the plasma membrane and is the source of energy for almost all other transporter proteins present in this membrane [5,6]. The energy generated by PM H^+^-ATPase enables plants to take up essential minerals from the soil against the gradient of their concentration [3]. PM H^+^-ATPase moves protons out from the cytoplasm to the apoplast, and consequently lowers the pH of the cell wall. This allows plant cells to expand according to the acid growth theory, in which acidification of the apoplast leads to the activation of enzymes responsible for loosening and modifying the cell wall structure [7]. In addition to lowering the apoplastic pH, PM H^+^-ATPase activity affects cell growth by hyperpolarising the cell membrane. This drives the increased influx of K^+^ ions, which changes the osmolality of the cell, resulting in water flow into the interior and increased turgor, involved in cell growth [3]. Modification of PM H^+^-ATPase activity may be crucial for maintaining the balance between plant growth processes and plant response to stress factors [8].

The basic component of living organisms is nitrogen (N). This is a part of amino acids, which are the building blocks of proteins. Plants absorb N from the soil in the form of ammonium or nitrate ions. Most plants use nitrate as a major source of nitrogen [9,10]. Nitrate transport is carried out by secondary symporters, which require a trans-membrane proton gradient previously generated by PM H^+^-ATPase. Two types of nitrate transport system function in plants: the low-affinity transport system (LATS) and the high-affinity transport system (HATS). LATS allows nitrate transport at high external NO_3_^−^ concentrations, whereas HATS provides nitrate uptake at low external NO_3_^−^ concentrations [9,11]. Nitrates taken from the soil solution are then reduced in cytosol to nitrite ions by nitrate reductase (NR) [12]. Subsequently, nitrite is reduced to ammonium ions by nitrite reductase (NiR) present in plastids. Finally, ammonium ions are incorporated into the amino acids via the GS-GOGAT cycle. The first step, i.e., the reduction of nitrates by NR, is a key moment in the assimilation of nitrates. For this reason, the activity of NR seems to be extremely important for plant growth processes.

Plant cell growth strictly depends on active phytohormone pools. Under unfavourable environmental conditions, the levels of these regulators change significantly in plant tissues. The survival of plants is related to their ability to adapt to a changing environment through complex signalling networks. Plant hormones play a major role in creating signal transduction responsible for the balance between plant growth and stress response. It was confirmed that jasmonates (JAs) are a crucial element in maintaining this balance under stressful conditions [13]. JAs are phytohormones derived from fatty acids that are found to modify plant growth. JAs include jasmonic acid (JA), its methyl ester (MeJA), and isoleucine conjugate (JA-Ile). They are generally considered to be stress hormones. It was shown that plants growing under disadvantageous conditions have increased levels of JAs in their tissues [14]. JA accumulation takes place in the cytosol, from where they are transported to the nucleus via a specific ABC protein (AtJAT1/AtABCG16), leading to changes in gene expression [15,16]. AtJAT1/AtABCG16 acts as a high-affinity transporter, which determines the subcellular distribution of JA. It is located both in the nuclear and plasma membranes of plant cells [17]. JAs are known to play an essential role in a plant’s response to pathogen attack, leading to the generation of reactive oxygen species (ROS) in plant cells [18]. Liu et al. [19] observed increased production of H_2_O_2_ in pea seedlings as a result of wounding. As a consequence, an increase in the JA level occurred in their tissues. ROS are one of the most common groups of toxic intermediates that are produced in plant cells under abiotic and biotic stresses. Among them, H_2_O_2_ is a dangerous metabolite due to the damage it can cause in cells. Originally, H_2_O_2_ was considered to be harmful to living organisms. However, this point of view has changed and the term double-faced molecule is often used; on the one hand, it is an oxidising element that is toxic to the cell, and on the other hand, it plays a very important signalling function by initiating signal transduction to protect plants against adverse environmental factors [20,21,22].

The aim of this study was to analyse the JA-dependent pathway leading to active growth inhibition in the roots of cucumber seedlings. Roots are the first organ to be exposed to unfavourable factors that enter a plant from the soil. Particular attention was paid to plasma membrane transport proteins that may be modulated by JA. For this reason, we examined both the activity of key growth factors, including PM H^+^-ATPase, nitrate transporters, and nitrate reductase, as well as the expression of genes encoding these proteins in roots of plants treated with JA. Since JA action is related to ROS generation in plant cells, we determined the changes in hydrogen peroxide levels and the expression of genes encoding enzymes involved in its production, i.e., RBOH and SOD. To our knowledge, the presented research is the first to comprehensively show the effects of jasmonic acid on the activity of proteins important for plant growth.

## 2. Materials and Methods

### 2.1. Plant Material

All experiments were performed on 6-day-old cucumber (*Cucumis sativus* L. cv. Wisconsin) seedlings. Cucumber seeds (from W. Legutko, Jutrosin, Poland) were germinated in darkness for 48 h at 27 °C and then transferred to a nutrient solution (1/3 strength Hoagland, pH 6.5) [23]. Plants were grown hydroponically under a 16 h photoperiod (180 mmol m^−2^ s^−1^) at 25 °C during the day and 22 °C during the night. For long JA and H_2_O_2_ treatments, different concentrations of JA or H_2_O_2_ (as indicated in the Figures) were added to the nutrient solution, and the seedlings were grown for 6 d. For the 24 h treatment, plants were first grown in a control nutrient solution (without the addition of JA/H_2_O_2_) for 5 d and then transferred to a fresh medium with the addition of different concentrations of JA or H_2_O_2_ or without these compounds (control) for the next 24 h. The roots, hypocotyls, and cotyledons were separated and weighed using an analytical balance (Ohaus AdventurerPro, accuracy 0.01 g; Ohaus, Nänikon, Switzerland) to quantify the fresh weight (FW). The lengths of the roots and hypocotyls were also measured. Most of the analyses, including enzymatic activity, gene expression, and uptake measurements, were performed on roots of seedlings treated with 1 µM JA for 24 h.

### 2.2. Determination of H_2_O_2_ and Lipid Peroxidation

The tissue content of H_2_O_2_ was quantified according to the method of Velikova et al. [24], with some modifications as described by Kabała et al. [25]. After incubation of the reaction mixture in darkness at room temperature for 60 min, the absorbance of triiodide (I_3_^−^), the product of potassium iodide (KI) oxidation, was measured at 390 nm.

The level of lipid peroxidation was measured according to Kabała et al. [26] with some modifications as described by Wdowikowska et al. [27]. In the method, the concentration of thiobarbituric acid reactive substances (TBARS), formed in acidic pH during incubation at 95 °C, was determined.

### 2.3. Isolation of Plasma Membrane and Determination of H^+^-ATPase Activities

Plasma membrane (PM) fraction was isolated according to the method of Larsson [28] with some modifications of Kłobus [29] using a 6.2% two-phase system containing PEG (polyethylene glycol) 3350 and dextran T500. The upper phase enriched in highly purified PM vesicles, right-side-out oriented, was collected and used for measurement of H^+^-ATPase hydrolytic activity. Some of PM vesicles were turned to the inside-out oriented form using Brij58 and used to determine ATP-dependent H^+^ transport across the PM. H^+^-ATPase hydrolytic activity was assayed by spectrophotometric measurement of the inorganic phosphate released from ATP according to Gallagher and Leonard [30]. To determine ATP-dependent H^+^ transport, changes in the absorbance of acridine orange at 495 nm (A_495_) were measured according to Kłobus and Buczek [31] and Janicka et al. [32]. Protein content was measured according to the method of Bradford [33]. Bovine serum albumin (BSA) was used as the standard.

### 2.4. Activities of NADPH-Generating Enzymes

The activities of four NADPH-generating enzymes, 6-phosphogluconate dehydrogenase (6PGDH, EC 1.1.1.44), glucose-6-phosphate dehydrogenase (G6PDH, EC 1.1.1.49), NADP-isocitrate dehydrogenase (NADP-ICDH, EC 1.1.1.42), and NADP-malic enzyme (NADP-ME, EC. 1.1.1.40), were measured according to Li et al. [34] using root extracts obtained from cucumber seedlings, as described by Jakubowska et al. [35]. NADPH production was expressed as the change in absorbance during NADP reduction, measured at 340 nm.

### 2.5. Nitrate Reductase Activity

Nitrate reductase (NR, EC 1.6.6.1) activity was measured in crude supernatants obtained from root tissues, according to the procedure described by Reda [36]. Reaction mixtures containing EDTA or Mg^2+^ were prepared according to Kaiser and Huber [37], with some modifications [36]. The amount of nitrite formed was measured colorimetrically with Griess reagent at 540 nm. NR activity is presented as NR total (NRtot) and NR actual (NRact) activities, determined in the presence of EDTA and Mg^2+^, respectively. The percentage of the unphosphorylated NR pool (dpNR) was estimated by calculating the NRact/NRtot ratio, and the phosphorylated NR pool (pNR) was valued using the formula pNR = 100 – (NRact/NRtot).

### 2.6. Nitrate Uptake Experiments

Cucumber seedlings were grown for 6 d on a nitrogen-free medium composed of 1 mM K_2_SO_4_, 0.2 mM Ca(H_2_PO_4_)_2_, 1.5 mM CaSO_4_, 0.33 mM MgSO_4_, 75 µM Fe-citrate, 10 µM MnSO_4_, 5 µM H_3_BO_4_, 1 µM CuSO_4_, 0.01 µM ZnSO_4_, and 0.05 µM Na_2_MoO_4_, pH 5.5. Then, the plants were transferred to an uptake solution, 10 mM Mes-NaOH pH 5.0 containing 0.7 mM CaSO_4_ and 0.5 mM KNO_3_, and incubated for 8 h with aeration. Every 2 h, the uptake solution was sampled to determine the amount of nitrate ions using an HPLC system with a Sphere-Image 80-5 SAX ion exchange column (Knauer, Berlin, Germany) [36]. Nitrate uptake was expressed as nitrate loss from the uptake solution.

### 2.7. Gene Expression Analysis

To evaluate gene expression, real-time PCR analysis was performed using a LightCycler 480 system (Roche, Basel, Switzerland). Total RNA was isolated from 70 mg of frozen powdered root tissue using EXRTAzol (Blirt, Gdansk, Poland). The concentrations and purity of the RNA preparations were determined with a NanoDrop Spectrophotometer ND-1000 (Thermo Fisher Scientific, Waltham, MA, USA). Samples showing 260/280 and 260/230 nm ratios of between 1.8 and 2.0 were purified from any DNA contamination with Rnase-free Dnase I (Fermentas, Waltham, MA, USA), and 2000 ng of RNA was used as a template for first-strand cDNA synthesis with the High-Capacity cDNA Reverse Transcription Kit (Applied Biosystems, Foster City, CA, USA). qRT-PCR was performed using a Real-Time 2 × PCR Master Mix SYBR kit (A&A Biotechnology, Gdańsk, Poland). Gene-specific primers for PCR amplification were used according to Wdowikowska and Kłobus (*CsHA*) [38], Jakubowska et al. (*CsRBOH)* [35], Reda et al. (*CsNR)* [39], and Kabała et al. (*CsCSD*, *CsMSD*, and *CsFSD)* [23]. For the normalisation of gene expression, *CsCACS* gene encoding the clathrin adaptor complex subunit [40] was used as an internal standard. Specific primer pairs for *CsNRT2*.1, *CsNRT2*.2, *CsNRT2*.3, and *CsNAR2* were designed, and the amplicons obtained were sequenced to confirm specificity of the PCR products. The sequences of all primers are listed in the Supplementary Materials (Table S1). The following conditions for qRT-PCR were applied: 30 s at 95 °C; 35–45 cycles of 10 s at 95 °C, 10 s at 56–62 °C, and 12 s at 72 °C; and final melting for 15 s at 65 °C.

### 2.8. Statistics

All presented data are the mean ± standard deviation (SD) from the number of experimental replications indicated in the Figure legends. qPT-PCR data were analysed by the ΔΔCT method using LightCycler software 4.1 (Roche, Basel, Switzerland). To compare the results to the controls, a one-sample *t*-test (*p* < 0.05) was used. For more than two groups of data, one-way ANOVA and Duncan’s post hoc analysis (*p* < 0.05) were used. All statistical analyses were performed with Statistica 13.3 (TIBCO Software Inc., Palo Alto, CA, USA).

## 3. Results

### 3.1. Effect of JA on the Growth of Cucumber Seedlings

Jasmonic acid (JA) has been found to modulate plant growth. Treatment of cucumber seedlings with this hormone for 6 d clearly affected plant weight and elongation (Figures S1A,B and S2), and the observed effects were dependent on JA concentration. Generally, lower JA levels, in the range of 50 nM to 1 µM, did not change significantly the weight and length of cucumber organs. In contrast, 10 µM and 50 µM JA decreased both growth parameters. The highest JA concentration (50 µM) showed the most pronounced effect. It reduced the weight and elongation of roots, hypocotyls, and cotyledons by about 40–60%. Notably, the highest root mass and hypocotyl length were found in plants treated with 1 µM JA.

When cucumber seedlings were exposed to JA for a shorter period (24 h), no significant changes in growth parameters were shown between control plants and plants treated with different concentrations of JA (Figure S3A,B). Based on the obtained results, 1 µM JA and a 24 h treatment time were selected for further analysis.

### 3.2. Effect of JA on H_2_O_2_ Level and Expression of Genes Involved in Its Regulation in Cucumber Roots

**Figure 1:**
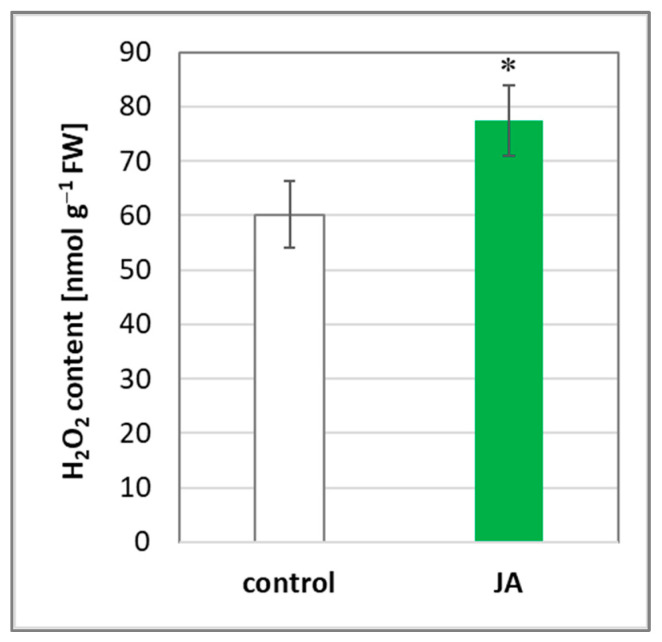
H_2_O_2_ level in cucumber roots. Plants were treated for 24 h with 1 µM JA or without this hormone (control). Results are means ± SD of three independent experiments run in triplicate. Statistically significant differences (*t*-test) between the control and treatment are marked by * (*p* < 0.05).

To explain the mode of JA action on cucumber seedlings, the level of H_2_O_2_, an ROS known to function as a signalling molecule, was determined in the roots of plants treated with 1 µM concentration of the hormone for 24 h. It was found that after exposure to JA, the H_2_O_2_ content increased by about 30% in the roots (Figure 1).

**Figure 2:**
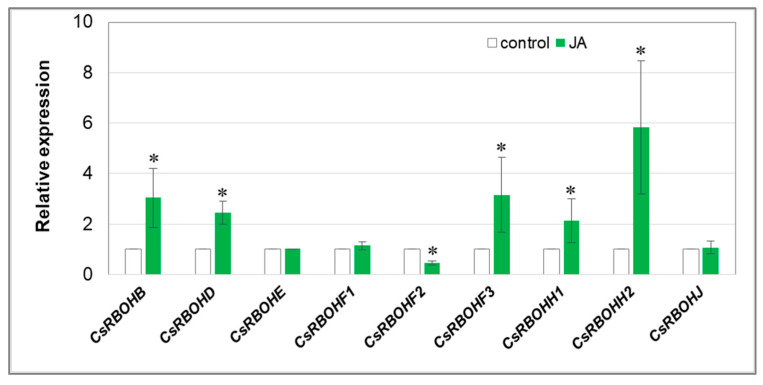
Relative expression level of *CsRBOH* genes in cucumber roots. Real-time PCR analysis was performed with the *CsCACS* reference gene used to normalise the results. RNA was isolated from the roots of control plants and plants treated with 1 µM JA for 24 h. Results are means ± SD of 4–6 replications of two independent experiments. Statistically significant differences (*t*-test) between the control and treatment are marked by * (*p* < 0.05).

The main source of ROS generated for signalling purposes appears to be plasma membrane-bound NADPH-oxidase (RBOH), involved in H_2_O_2_ formation in apoplast. Exposure of seedlings to 1 µM JA significantly activated the expression of five *CsRBOH* genes in roots (Figure 2). The JA-induced increase in the expression of *CsRBOHB*, *CsRBOHD*, *CsRBOHF3*, *CsRBOHH1*, and *CsRBOHH2* was 3, 2.5, 3.2, 2.1, and 5.8 times higher, respectively, than in the roots of the control plants. The increase in the transcript level seems to be particularly significant in the case of *CsRBOHB* and *CsRBOHD*, which are most highly expressed in cucumber roots (Table S2).

**Figure 3:**
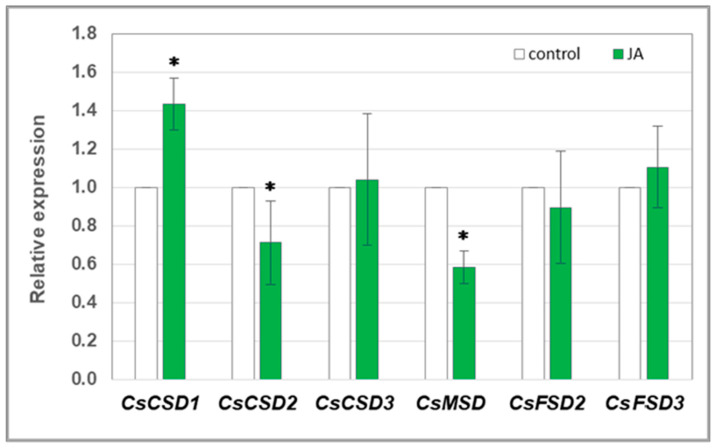
Relative expression level of genes encoding SOD isoforms in cucumber roots. Real-time PCR analysis was performed with the *CsCACS* reference gene used to normalise the results. RNA was isolated from the roots of control plants and plants treated with 1 µM JA for 24 h. The results are means ± SD of six replications of two independent experiments. Statistically significant differences (*t*-test) between the control and treatment are marked by * (*p* < 0.05).

Since the activity of plasma membrane NADPH oxidase is strictly related to the action of superoxide dismutase (SOD), the expression level of genes encoding individual dismutase isoforms was analysed in cucumber roots exposed to JA. It was indicated that the expression of one of them, *CsCSD1*, was upregulated in seedlings treated with 1 µM JA (Figure 3). In contrast, the transcript levels of *CsCSD2* and *CsMSD* significantly decreased in roots after JA exposure. *CsCSD1* is the most highly expressed SOD-encoding gene in cucumber roots (Table S3).

### 3.3. Effect of JA on the Activity of NADPH-Producing Enzymes

**Figure 4:**
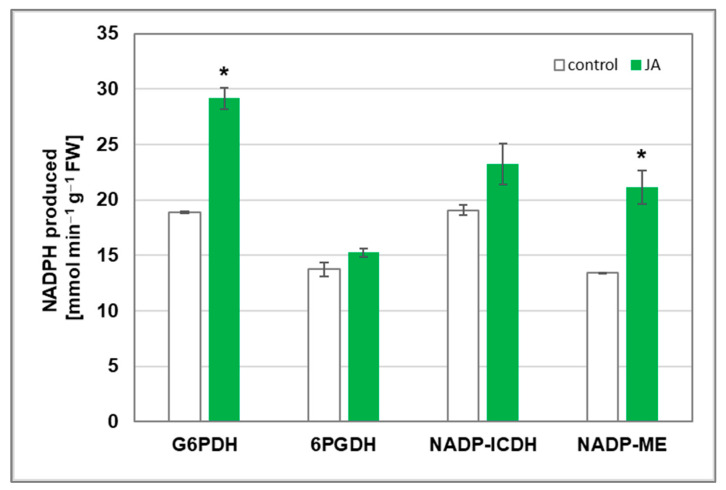
Activity of enzymes involved in NADPH generation in cucumber roots. Plants were treated for 24 h with 1 µM JA or without this hormone (control). Presented results are means ± SD of two independent experiments, each run in triplicate. Statistically significant differences (*t*-test) between the control and treatment are marked by * (*p* < 0.05).

On the other hand, NADPH oxidase action depends on its metabolic substrate, i.e., NADPH. For this reason, the levels of four NADPH-producing enzymes, including glucose-6-phosphate dehydrogenase (G6PDH), 6-phosphogluconate dehydrogenase (6PGDH), NADP-isocitrate dehydrogenase (NADP-ICDH), and NADP-malic enzyme (NADP-ME), were measured in cucumber seedlings treated with 1 µM JA (Figure 4). Both G6PDH and NADP-ME were found to be significantly stimulated by this hormone. The enzyme activities reached about 155% of the control value in roots. In contrast, the activities of 6PGDH and NADP-ICDH remained relatively unaffected by JA, although their elevated level has also been demonstrated.

### 3.4. Effect of H_2_O_2_ on the Growth of Cucumber Seedlings

Endogenous H_2_O_2_ increased as a result of plant treatment with JA; therefore, in the next step, the effect of exogenous hydrogen peroxide on the growth parameters of cucumber seedlings was verified. The plants were treated with three different concentrations of H_2_O_2_ for 6 d (Figure S4). 5 mM H_2_O_2_ was shown to significantly inhibit cucumber organ growth, reducing root and cotyledon weight by 50% and 37%, respectively (Figure S4A). Similarly, the length of the roots and hypocotyls decreased, reaching approximately 50% and 70%, respectively, compared to the control (Figure S4B). The other H_2_O_2_ concentrations used had no significant effect on the growth of cucumber seedlings, with the exception of 1 mM, which increased the weight of hypocotyls.

On the other hand, exposure of seedlings to H_2_O_2_ for 24 h did not affect the growth of hypocotyls and cotyledons. However, a reduction in root weight was observed. All H_2_O_2_ concentrations decreased this parameter to the same extent, by 21–24% (Figure S5A,B).

### 3.5. Effect of JA on Lipid Peroxidation in the Roots of Cucumber Seedlings

**Figure 5:**
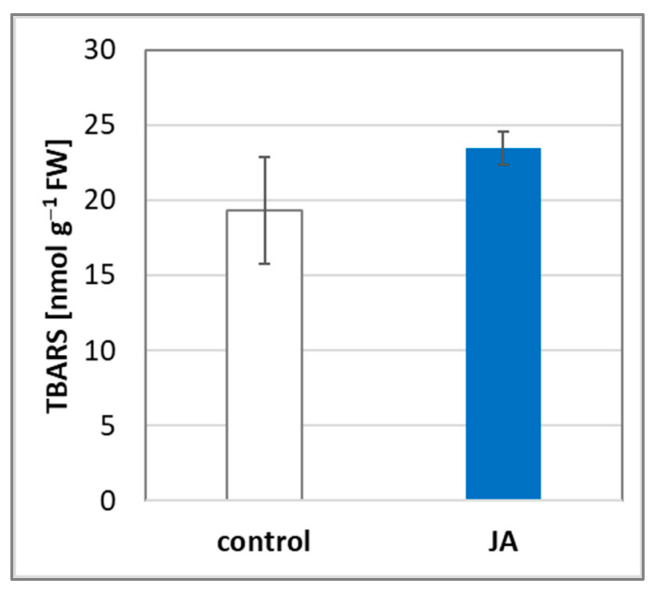
TBARS level in cucumber roots. Plants were treated for 24 h with 1 µM JA or without this hormone (control). Results are means ± SD of three independent experiments run in triplicate. There were no statistically significant differences between control and treatment (*t*-test, *p* < 0.05).

The increase in H_2_O_2_ content, observed after plant treatment with JA, may be related to the induction of oxidative stress. Therefore, the level of lipid peroxidation, which is an indicator of oxidative damage, was measured in the roots of plants exposed to this hormone for 24 h (Figure 5). The results indicated that JA does not affect lipid peroxidation, suggesting that oxidative changes are not activated.

### 3.6. Regulation of the Plasma Membrane Proton Pump by JA in the Roots of Cucumber Seedlings

**Figure 6:**
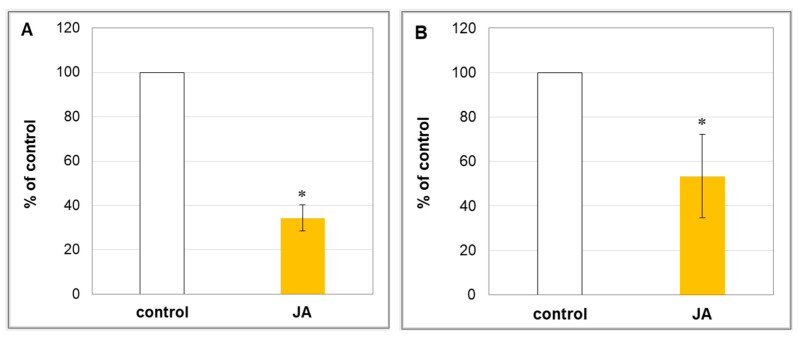
Plasma membrane H^+^-ATPase activity, measured as ATP hydrolysis (**A**) and ATP-driven proton transport (**B**), in cucumber roots. Plants were treated for 24 h with 1 µM JA or without this hormone (control). Results are means ± SD of 3–4 independent experiments run in triplicate. Hydrolytic activity of H^+^-ATPase reached 758 µg Pi h^−1^ mg^−1^ protein in control samples (100%). Mean ATP-dependent H^+^ transport of control samples was 2.068 A_495_ min^−1^ mg^−1^ protein (100%). Statistically significant differences (*t*-test) between the control and treatment are marked as * (*p* < 0.05).

Plasma membrane H^+^-ATPase is a proton pump involved in the growth processes of plant cells. For this reason, the activity of this enzyme was analysed in cucumber seedlings treated with JA for 24 h. JA was shown to inhibit both ATP hydrolysis (Figure 6A) and ATP-dependent proton transport (Figure 6B) in plasma membrane vesicles isolated from roots. Hydrolytic activity reached 35% of the control level, while H^+^ pumping was diminished to about 50% of the control value.

**Figure 7:**
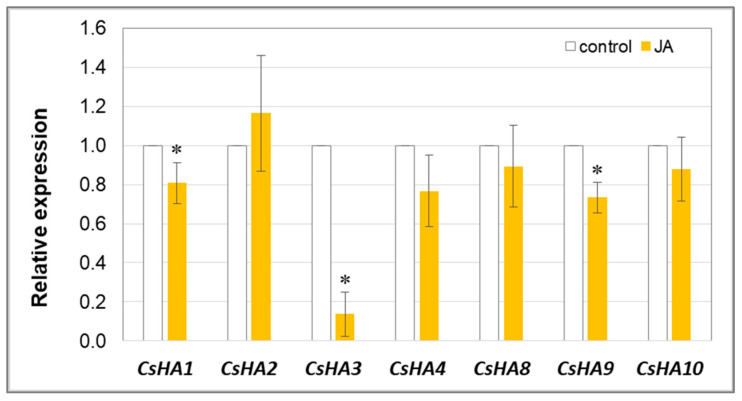
Expression level of *CsHA* genes in cucumber roots. Real-time PCR analysis was performed with the *CsCACS* reference gene used to normalise the results. RNA was isolated from the roots of control plants and plants treated with 1 µM JA for 24 h. Results are means ± SD of 4–6 replications of two independent experiments. Statistically significant differences (*t*-test) between the control and treatment are marked by * (*p* < 0.05).

The decrease in H^+^-ATPase activity was related to the downregulation of gene expression (Figure 7). Three of the seven genes encoding enzyme isoforms (*CsHA1*, *CsHA3*, and *CsHA9*) showed reduced transcription levels in the roots of plants treated with JA for 24 h. Among them, *CsHA3* expression decreased the greatest extent, reaching 14% of the level determined in control roots. *CsHA3* is a unique gene among all *CsHAs*. It is expressed at high levels exclusively in cucumber roots (Table S4).

### 3.7. Regulation of Nitrate Uptake and Assimilation by JA in the Roots of Cucumber Seedlings

**Figure 8:**
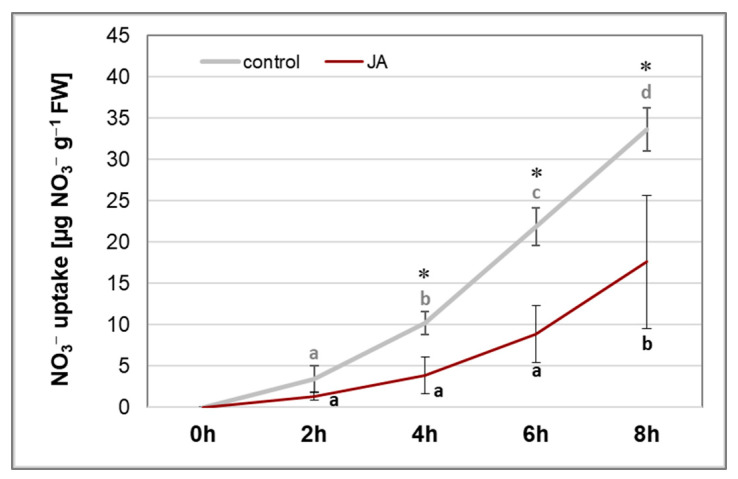
Nitrate uptake by cucumber roots. Plants were treated for 24 h with 1 µM JA or without this hormone (control). After treatment, plants were incubated with 0.5 mM KNO_3_ in Mes-NaOH (pH 5.5). Data are the means of at least six biological repetitions. Different letters represent homogeneous groups, independently in control plants and JA-treated plants, according to Duncan’s test (*p* < 0.05). Statistically significant differences (*t*-test) between the control and treatment in the same time interval are marked as * (*p* < 0.05).

The proton gradient, generated by the plasma membrane H^+^-ATPase, energises secondary active symporters responsible for the nutrient uptake by root cells. It was found that application of 1 µM JA significantly inhibits nitrate uptake by cucumber seedlings (Figure 8). In the control plants, it was found that the roots took up nitrates throughout the measurement period, i.e., up to 8 h. In contrast, in plants treated for 24 h with JA, no significant changes in the level of nitrates absorbed within 6 h were found. In these seedlings, the total amount of nitrates taken up for 8 h reached about 50% of the level determined in the roots of control plants.

**Figure 9:**
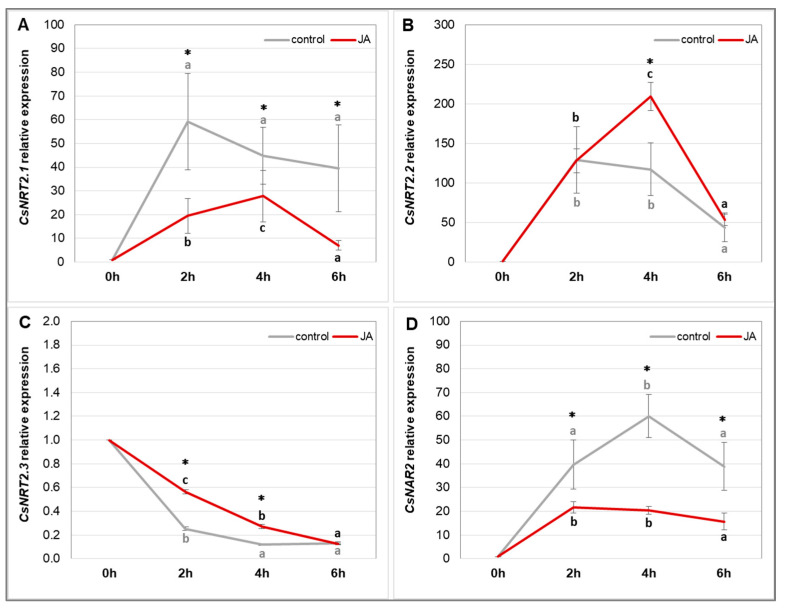
Relative expression level of *CsNRT2.1* (**A**), *CsNRT2.2* (**B**), *CsNRT2.3* (**C**), and *CsNAR2* (**D**) genes in cucumber roots. Real-time PCR analysis was performed with the *CsCACS* reference gene used to normalise the results. RNA was isolated from the roots of control plants and plants treated with 1 µM JA for 24 h. After treatment, plants were incubated with 0.5 mM KNO_3_ in Mes-NaOH (pH 5.5). Data are the means of at least three replications of two biological repetitions. Different letters represent homogeneous groups, independently in control plants and JA-treated plants, according to Duncan’s test (*p* < 0.05). Statistically significant differences (*t*-test) between the control and treatment in the same time interval are marked by * (*p* < 0.05).

Analysis of the expression level of four genes encoding proteins participating in high-affinity nitrate uptake by cucumber roots indicated that they are differently regulated by 1 µM JA (Figure 9). JA was found to significantly inhibit the transcription of *CsNRT2.1* encoding a high-affinity NO_3_^−^ transporter (Figure 9A) and *CsNAR2* encoding a regulatory protein necessary for proper functioning of NRT2 transporters (Figure 9D). On the other hand, the expression of two other genes involved in nitrate absorption, *CsNRT2.2* and *CsNRT2.3*, increased after plants were treated with JA, but at 6 h it did not differ from the control (Figure 9B,C).

**Figure 10:**
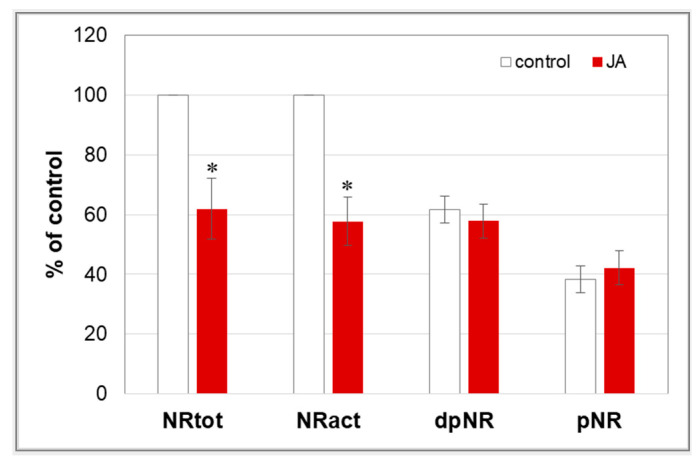
Nitrate reductase (NR) activity in cucumber roots. Plants were treated for 24 h with 1 µM JA or without this hormone (control). NR activity was expressed as total (NRtot) and actual (NRact) activity according to the Materials and methods. Based on NRtot and NRact values, the level of unphosphorylated (dpNR) and phosphorylated (pNR) forms of NR was estimated. Results are means ± SD of three independent experiments run in triplicate. Some 904 (NRtot) and 547 (NRact) nmoles of NO_2_^−^ h^−1^ g^−1^ FW was considered as 100%. Statistically significant differences (*t*-test) between the control and treatment are marked by * (*p* < 0.05).

The first step in nitrate assimilation involves the activity of nitrate reductase (NR). Due to post-translational regulation of NR activity via phosphorylation, total (NRtot) and actual (NRact) enzyme activity was determined. NRtot expresses the activity of both the phosphorylated (pNR) and unphosphorylated (dpNR) forms of NR. NRact indicates activity in only the dpNR form. The results showed that both NRtot and NRact enzyme activities decreased by approximately 40% in the roots of cucumber seedlings treated with 1 µM JA (Figure 10). In contrast, the phosphorylation status of NR did not change after plant treatment with JA. Similar levels of the pNR and dpNR forms of the enzyme were found.

**Figure 11:**
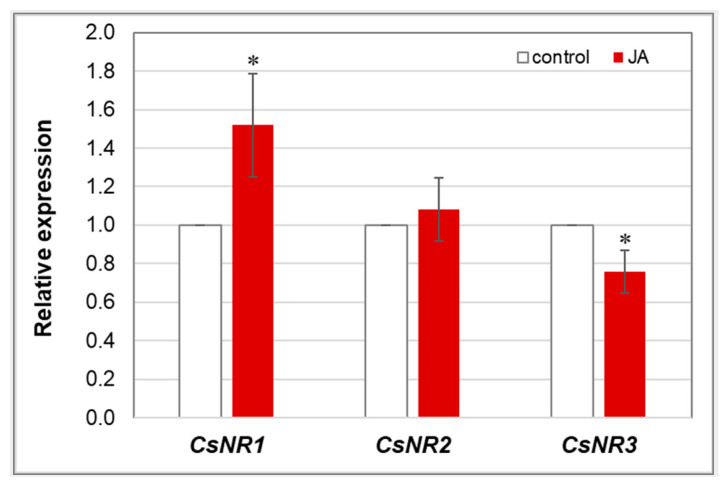
Relative expression level of *CsNR* genes in cucumber roots. Real-time PCR analysis was performed with the *CsCACS* reference gene used to normalise the results. RNA was isolated from the roots of control plants and plants treated with 1 µM JA for 24 h. Results are means ± SD of six replications of two independent experiments. Statistically significant differences (*t*-test) between the control and treatment are marked by * (*p* < 0.05).

The JA-induced decrease in NR activity was not strictly related to gene expression level. Transcription of only one (*CsNR3*) of the three genes, encoding NR in cucumber roots, was downregulated (Figure 11). In contrast, JA enhanced the expression of *CsNR1* and had no effect on the expression level of *CsNR2*. Both genes of which transcription was affected by JA (*CsNR1* and *CsNR3*) are expressed at much lower levels than *CsNR2* in cucumber roots (Table S5).

## 4. Discussion

An increase in the level of various plant hormones, both growth stimulators and inhibitors, is often observed in plants exposed to abiotic and biotic stresses. It is therefore interesting and noteworthy to understand the role of individual phytohormones for the functioning of plants under unfavourable conditions. Usually, when environmental conditions are not optimal, plant growth is weakened [1]. Plants therefore need to modify their metabolism and switch to fight the stressor, protecting themselves from adverse factors until they subside. During this time, plants cannot waste energy and metabolic resources on growth processes. Active growth retardation allows plants to defend themselves against unfavourable environmental conditions [1]. Because active growth inhibition is beneficial for plant survival, we analysed this strategy in the context of jasmonates (JAs), widely considered as growth inhibitors. It was found that JAs, which are lipid-derived stress phytohormones, inhibit plant growth and reduce meristem activity [41,42,43]. In our studies, we also observed that JA treatment inhibits the growth of cucumber seedlings (Figures S1A,B and S2). Moreover, other studies have shown that jasmonates inhibit root elongation by limiting cell enlargement [44]. On the other hand, in plants growing under unfavourable conditions, an increased level of JAs was demonstrated [14,45]. This accumulation occurs in the cytosol, from where JAs enter the cell nucleus and can modify the expression of appropriate genes, supporting defence reactions [16]. JA signalling is a major stress hormone pathway that interacts with other phytohormones to create a complex network [46]. Negative crosstalk between growth stimulators and JA, for example, cytokinin-JA [43], auxins-JA [47,48], and gibberellins-JA [49,50], was identified most often. Such hormonal interactions may be responsible for limiting plant growth. To date, there are no scientific reports that comprehensively investigate the involvement of jasmonic acid in inhibiting plant growth. Therefore, we verified the effect of JA on cucumber seedlings by analysing the elements important for the physiology and metabolism of plant cells, crucial for the growth process. In our research, we noticed that JA inhibited the growth of both the aboveground and underground parts of cucumber seedlings (Figure S1A,B). At higher concentrations of JA, a more visible growth inhibition (both weight and length) was observed when plants were treated with this hormone for 6 d. This effect was not evident in plants exposed to JA for 24 h (Figure S3A,B), probably due to too short a time to observe significant changes in basic growth parameters. It seems, however, that in the first hours after the application of JA to cucumber plants, there were some changes in cell metabolism, the consequence of which was a visible inhibition of growth observed after 6 d. For this reason, we focused on analysing the changes occurring after 24 h of plant treatment with JA.

One of the effects of JA action on plants is enhanced production of reactive oxygen species, found in host cells after pathogen attack. The oxidative burst, i.e., the production of ROS by oxygen consumption, is a rapid response of plant cells. Many reports have indicated generation of apoplastic superoxide or its dismutation product, hydrogen peroxide, after a pathogen attack [18,51]. Under such conditions, the most studied reactive oxygen species is H_2_O_2_, which is more stable than other ROS [52]. Therefore, it seemed important to investigate whether the observed JA-induced growth inhibition results from the activation of signalling pathways involving H_2_O_2_. First, we observed a significant increase in H_2_O_2_ level in root tissues of cucumber seedlings treated with 1 µM JA for 24 h (Figure 1). Our results are consistent with those of Liu et al. [19], who observed increased production of H_2_O_2_ in pea (*Pisum sativum* L.) seedlings as a result of wounding. An increase in JA level was positively correlated with an increase in hydrogen peroxide level. Similarly, Hung et al. [53] demonstrated a relation between jasmonates and H_2_O_2_. The treatment of rice leaves with methyl jasmonate caused an increase in H_2_O_2_ content. Furthermore, in our study, it was shown that exposure of cucumber plants to H_2_O_2_ significantly inhibited their growth (Figures S4 and S5). ROS are believed to be the main element of signalling network activated in plants growing under unfavourable conditions. They interact with other secondary messengers and plant hormones, enabling signal transmission [54].

To pinpoint the source of the hydrogen peroxide observed in JA-treated plants, the transcript level of some genes related to ROS metabolism was analysed. We showed that JA increased the expression of several isoforms of the genes encoding both RBOH and SOD, which are proteins involved in ROS production. H_2_O_2_ formed as a result of their action is located primarily near the plasma membrane, in the apoplast. RBOH contributes to the production of superoxide anion radicals, which are immediately decomposed by SOD into a less harmful form of hydrogen peroxide. Many scientific reports have indicated that JAs contribute to the increase in SOD activity in plant tissues treated with this phytohormone [55,56,57,58,59]. We observed JA-dependent upregulation of genes encoding five isoforms of plasma membrane NADPH oxidase in roots of cucumber seedlings: *CsRBOHB*, *CsRBOHD*, *CsRBOHF3*, *CsRBOHH1*, and *CsRBOHH2* (Figure 2). Two of them, *CsRBOHB* and *CsRBOHD,* are mostly expressed in the roots (Table S2). The role of NADPH oxidase in H_2_O_2_ generation in plants treated with JAs was demonstrated by Maruta et al. [60]. By characterising single knockout mutants for all AtRBOH proteins, they found that two isoforms, AtRBOHD and AtRBOHF, are particularly involved in ROS production in response to plant spraying with 50 µM MeJA solution. On the other hand, among the analysed genes encoding different SOD isoforms, i.e., CSD (Copper–Zinc SODs, Cu/Zn-SOD), MSD (Manganese SODs, Mn-SOD), and FSD (Iron SODs, Fe-SOD), only the expression of *CsCSD1* was significantly increased (Figure 3). Importantly, the *CsCSD1* gene shows the highest transcript level in the roots of cucumber seedlings under control conditions (Table S3). Cu/Zn-SOD is the only one of the three dismutase groups that is located extracellularly, in the apoplast [61,62]. Therefore, it seems to be no coincidence that the *CsCSD1* gene encoding the isoform, the presence of which is probably extracellular, was upregulated in cucumber seedlings treated with JA. It is believed that hydrogen peroxide present in the apoplast plays a signalling role and is less toxic than its counterpart in other cell spaces, such as the cytosol, chloroplasts, and nucleus. In these compartments, H_2_O_2_ can lead to drastic changes, causing oxidative stress and damage of various macromolecules (proteins, DNA, RNA, and lipids). Additionally, an increase in apoplastic ROS production has been shown to participate in cell signalling through the regulation of nuclear gene transcription [63,64]. The less harmful effect of H_2_O_2_ produced in the apoplast can also be confirmed by the results of our experiments demonstrating the TBARS levels in cucumber roots. The TBARS assay is widely used to measure lipid peroxidation in biological samples and is a good indicator of oxidative stress [65]. Despite the increase in hydrogen peroxide, we did not observe significant changes in lipid peroxidation in cucumber seedlings treated with JA (Figure 5). This suggests that oxidative changes do not occur in cells of roots exposed to JA. Sirhindi et al. [66], who examined stress markers in *Glycine max* seedlings, also observed no changes in TBARS level when plants were treated with 1 µM JA for 6 h.

In addition to modifying gene expression, JA may contribute to the stimulation of the PM NADPH oxidase by increasing the cytosolic amount of substrate needed for its activity. In cucumber seedlings treated with JA, an increase in the activity of the enzymes involved in NADPH production was observed (Figure 4). Such conditions may favour upregulation of plasma membrane NADPH oxidase. RBOH activity requires NADPH in the cytosol, which is supplied mainly by four enzymes: G6PDH, 6PGDH, NADP-ME, and NADP-ICDH [35]. JA strongly stimulates G6PDH and NADP-ME activities in roots of cucumber seedlings. G6PDH is a component of the oxidative pentose phosphate pathway (OPPP), which is the main source of reducing power in plant cells. In addition, studies have shown that this enzyme plays an important role during abiotic stress [67]. Moreover, NADP-ME has been found to be an important producer of NADPH when plants are exposed to abiotic stresses, thus supporting OPPP [68].

The main question posed in this work was why jasmonates affect growth processes. We examined the behaviour of plasma membrane proton pump (PM H^+^-ATPase), which is crucial for plant growth, in roots of JA-treated plants. There are no data on the JA influence on the activity of this key enzyme. Under physiological conditions, the main function of PM H^+^-ATPase is to generate the energy necessary for secondary transport [3]. Moreover, the action of PM H^+^-ATPase causes a decrease in the pH of cell walls. This allows plant cells to expand according to the acid growth theory. In addition, PM H^+^-ATPase activity affects cell growth by hyperpolarising the cell membrane. This leads to an increased import of K^+^ ions, resulting in an influx of water into the cell and an increase in turgor, which contributes to cell expansion [3]. JA was shown to inhibit the activity of plasma membrane proton pump in cucumber roots (Figure 6). This can be crucial in switching from a “growth” strategy to a “defence” strategy. The inhibition of the activity of this enzyme reduces energy consumption. A smaller pool of ATP is hydrolysed, and the substrates are not used as plant building materials, but they can be redirected to the formation of appropriate proteins or defensive/protective compounds. The inhibited activity of the PM H^+^-ATPase may be at least partially related to the downregulation of the expression of gene encoding the CsHA3 protein, which is the most abundant proton pump isoform next to CsHA2 in the roots of cucumber seedlings (Figure 7, Table S4). Other results were obtained by Zhu et al. [69] and Yan et al. [59] using exogenous application of MeJA. The efflux of protons from cells, acidification of the apoplast, and an increase in the hydrolytic activity of the pump have been observed in the roots of lettuce seedlings exposed to MeJA [69]. In *Arabidopsis* guard cells, MeJA treatment resulted in an increase in cytosolic pH and H^+^ efflux from cells. However, these effects did not occur in the presence of orthovanadate (PM H^+^-ATPase inhibitor) or in the mutant defective in one of the PM H^+^-ATPase genes, *AHA1* [59]. These discrepancies may be due to the different forms of jasmonates used in the studies; we used jasmonic acid (JA), whereas Zhu et al. [69] and Yan et al. [59] used methyl jasmonate. On the other hand, injured tomato plants showed an increase in JA level and, at the same time, inhibition of plasma membrane proton pump was noted [70]. This is consistent with the results of our study. The JA-dependent inhibition of PM H^+^-ATPase observed in cucumber seedlings can result not only from JA-induced downregulation of *CsHA3* gene expression by this hormone but also from the action of H_2_O_2_. In our previous study, treatment of cucumber seedlings for 24 h with different concentrations of hydrogen peroxide (0.1–20 mM) inhibited both the hydrolytic activity and proton transport generated by PM H^+^-ATPase [71]. This means that maintaining elevated levels of hydrogen peroxide in plants during the initial period of the stress reaction may be a beneficial element in the growth inhibition strategy by reducing pump activity due to the operation of reactive oxygen species. The action of JA in the “switch growth to defence” strategy seems to not only increase the level of hydrogen peroxide but also maintain it at the appropriate level in plant cells. According to this, JA did not cause any changes in catalase activity in roots of cucumber seedlings treated for 24 h with 1 µM JA [72]. However, we did not exclude the fact that other enzymes (for example, POX) or non-enzymatic antioxidants may also be involved in this process.

For plant growth processes, uptake of nutrients is very important. Among them, nitrates, which are a source of nitrogen for amino acids that have formed, and, consequently, proteins play an essential role in building a growing organism. Nitrates are reduced to nitrites by nitrate reductase (NR) and further to ammonium by nitrite reductase before being incorporated into amino acids [9]. Therefore, inhibition of nitrate uptake and assimilation is another factor limiting plant growth. Nitrate transport activity is coupled to the electrochemical proton gradient generated by plasma membrane proton pump [10]. Treatment of cucumber seedlings with 1 µM JA significantly inhibited nitrate absorption by roots (Figure 8). Similarly, in rice, a drastic reduction in nitrate uptake was observed as a result of plant exposure to MeJA [73]. The reduction of nitrate uptake in JA-treated plants, observed in our study, may be due to the inhibition of PM H^+^-ATPase activity, thereby preventing the functioning of active nitrate symporters. On the other hand, this inhibition may be related to the modification of the expression of genes encoding nitrate transporters in cucumber roots. It was shown that JA reduced the transcript level of *CsNRT2.1* encoding the main nitrate transporter belonging to HATS (Figure 9A). In contrast, we observed an increase in the expression of *CsNRT2.2* and *CsNRT2.3* genes after JA exposure (Figure 9B,C). However, this effect does not seem to be important for nitrate uptake because of the NAR protein, which is crucial for the function of nitrate transporters. The *NAR2*-type genes encode small proteins that are part of a two-component nitrate high-affinity transport system. Interaction with the NAR protein is essential for nitrate uptake by some NRT2s [74]. Lupini et al. [75] showed that *ZmNAR2.1* plays a significant role in the regulation of *ZmNRT2.1* expression and this is correlated with modulation of nitrate influx. In cucumber seedlings, JA caused the inhibition of *CsNAR2* expression (Figure 9D), suggesting that this effect may be responsible for the limited nitrate uptake. Moreover, we observed an interesting phenomenon with *CsNRT2.3* gene expression. CsNRT2.3 protein belongs to the HATS transporter family and its expression is repressed when nitrates appear in nutrient solution [76]. Our results showed that the transcript level of *CsNRT2.3* decreased due to the addition of nitrates into the growing medium in roots of both control and JA-treated plants. However, the decrease in *CsNRT2.3* expression was greater in the control than in the JA-exposed plants. It seems very likely that nitrate uptake was more efficient in the control, and more ions entered the cells. When nitrates enter the cells, the expression of *CsNRT2.3* is inhibited. In JA-treated plants, the uptake of nitrates is weaker due to the inhibited proton pump and, as a consequence, the decrease in *CsNRT2.3* expression is slower. 

Once absorbed, nitrate must be reduced to ammonia in plant tissues before it can be incorporated into amino acids. NR is an enzyme that catalyses the first step of nitrate assimilation, reducing nitrates to nitrites [77]. It is a substrate-induced enzyme; therefore, a limited pool of nitrates in the cytosol inhibits its activity [12]. Our studies have shown that NR activity decreases in the roots of cucumber seedlings treated with JA (Figure 10). The observed inhibition was probably due to the reduced nitrate uptake by cucumber seedlings. Moreover, reduced expression of *CsNR3* in JA-treated cucumber roots may have contributed to the lower NR activity. On the other hand, among the three isoforms of genes encoding nitrate reductase in cucumber, the expression of one, *CsNR1*, was slightly increased by JA. It is worth noticing that the transcript levels of *CsNR1* and *CsNR3* isoforms were several times lower than that of the dominant *CsNR2*, whose expression was unchanged in plants treated with JA (Table S5). There are no other scientific reports showing JA effects on the activity of NR in plants. However, the application of MeJA enhanced NR activity in *Glycyrrhiza uralensis* [78]. Interestingly, as previously indicated, the use of a different form of jasmonates, MeJA, had the opposite effect on the activity of the studied protein than JA. The diminished reduction of nitrates, due to the inhibition of NR activity, may consequently result in a reduction in the level of proteins in tissues, thus limiting the growth of cucumber seedlings. For example, treatment of soybeans with JA resulted in reduced protein content [79].

## 5. Conclusions

In summary, in this study, we have analysed for the first time the key elements responsible for growth processes, including PM H^+^-ATPase activity, nitrate transport, and nitrate reductase activity, in plants treated with jasmonic acid. We have shown that JA contributed to an increase in hydrogen peroxide level in roots by enhancing the expression of genes encoding RBOH and SOD. Jasmonic acid, alone or via hydrogen peroxide, inhibited plasma membrane proton pump activity, resulting in reduced nitrate uptake and altering nitrate reductase activity. We believe that the sequence of events demonstrated in JA-treated cucumber plants leads to the active inhibition of growth, allowing plants to switch to a strategy of fighting against adverse environmental factors. It will be interesting to verify in future studies whether active growth inhibition is similar or different depending on the jasmonate form used. Based on the research conducted as part of this work and other scientific reports, it can be assumed that this reaction may be specific

## Data Availability

The data presented are available in this manuscript and Supplementary Materials.

## Supplementary Materials

The following supporting information can be downloaded at: https://www.mdpi.com/article/10.3390/cells12182263/s1. Figure S1: Weight (A) and length (B) of individual organs of cucumber seedlings exposed to jasmonic acid (JA) for 6 d. Figure S2: Representative image of cucumber seedlings treated with different concentrations of jasmonic acid (JA) for 6 d. Figure S3: Weight (A) and length (B) of individual organs of cucumber seedlings exposed to jasmonic acid (JA) for 24 h. Figure S4: Weight (A) and length (B) of individual organs of cucumber seedlings exposed to H_2_O_2_ for 6 d. Figure S5: Weight (A) and length (B) of individual organs of cucumber seedlings exposed to H_2_O_2_ for 24 h. Table S1: The primers used in qRT-PCR analysis; Table S2: Transcript level of *CsRBOH* genes in roots of cucumber control plants; Table S3: Transcript level of genes encoding SOD isoforms in roots of cucumber control plants; Table S4: Transcript level of *CsHA* genes in roots of cucumber control plants; Table S5: Transcript level of *CsNR* genes in roots of cucumber control plants.

## References

[1] Zhang H., Zhao Y., Zhu J.-K. (2020). Thriving under Stress: How Plants Balance Growth and the Stress Response. Dev. Cell.

[2] Kabała K., Janicka M. (2023). Structural and Functional Diversity of Two ATP-Driven Plant Proton Pumps. Int. J. Mol. Sci..

[3] Morsomme P., Boutry M. (2000). The Plant Plasma Membrane H^+^-ATPase: Structure, Function and Regulation. Biochim. Biophys. Acta Biomembr..

[4] Arango M., Gévaudant F., Oufattole M., Boutry M. (2003). The Plasma Membrane Proton Pump ATPase: The Significance of Gene Subfamilies. Planta.

[5] Falhof J., Pedersen J.T., Fuglsang A.T., Palmgren M. (2016). Plasma Membrane H^+^-ATPase Regulation in the Center of Plant Physiology. Mol. Plant.

[6] Duby G., Boutry M. (2009). The Plant Plasma Membrane Proton Pump ATPase: A Highly Regulated P-Type ATPase with Multiple Physiological Roles. Pflugers Arch..

[7] Arsuffi G., Braybrook S.A. (2018). Acid Growth: An Ongoing Trip. J. Exp. Bot..

[8] Janicka-Russak M. (2011). Plant Plasma Membrane H^+^-ATPase in Adaptation of Plants to Abiotic Stresses. Abiotic Stress Response in Plants–Physiological, Biochemical and Genetic Perspectives.

[9] Noguero M., Lacombe B. (2016). Transporters Involved in Root Nitrate Uptake and Sensing by *Arabidopsis*. Front. Plant Sci..

[10] Fan X., Naz M., Fan X., Xuan W., Miller A.J., Xu G. (2017). Plant Nitrate Transporters: From Gene Function to Application. J. Exp. Bot..

[11] Miller A.J., Fan X., Orsel M., Smith S.J., Wells D.M. (2007). Nitrate Transport and Signalling. J. Exp. Bot..

[12] Reda M., Klobus G. (2006). Modifications of the Activity of Nitrate Reductase from Cucumber Roots. Biol. Plant.

[13] Ruan J., Zhou Y., Zhou M., Yan J., Khurshid M., Weng W., Cheng J., Zhang K. (2019). Jasmonic Acid Signaling Pathway in Plants. Int. J. Mol. Sci..

[14] De Domenico S., Taurino M., Gallo A., Poltronieri P., Pastor V., Flors V., Santino A. (2019). Oxylipin Dynamics in *Medicago truncatula* in Response to Salt and Wounding Stresses. Physiol. Plant.

[15] Gupta A., Hisano H., Hojo Y., Matsuura T., Ikeda Y., Mori I.C., Senthil-Kumar M. (2017). Global Profiling of Phytohormone Dynamics during Combined Drought and Pathogen Stress in *Arabidopsis thaliana* Reveals ABA and JA as Major Regulators. Sci. Rep..

[16] Ali M.S., Baek K.-H. (2020). Jasmonic Acid Signaling Pathway in Response to Abiotic Stresses in Plants. Int. J. Mol. Sci..

[17] Li Q., Zheng J., Li S., Huang G., Skilling S.J., Wang L., Li L., Li M., Yuan L., Liu P. (2017). Transporter-Mediated Nuclear Entry of Jasmonoyl-Isoleucine Is Essential for Jasmonate Signaling. Mol. Plant.

[18] Auh C.K., Murphy T.M. (1995). Plasma Membrane Redox Enzyme Is Involved in the Synthesis of O_2_^-^ and H_2_O_2_ by Phytophthora Elicitor-Stimulated Rose Cells. Plant Physiol..

[19] Liu Y., Pan Q.H., Yang H.R., Liu Y.Y., Huang W.D. (2008). Relationship between H_2_O_2_ and Jasmonic Acid in Pea Leaf Wounding Response. Russ. J. Plant Physiol..

[20] Suzuki N., Mittler R. (2006). Reactive Oxygen Species and Temperature Stresses: A Delicate Balance between Signaling and Destruction. Physiol. Plant.

[21] Volkov R.A., Panchuk I.I., Mullineaux P.M., Schöffl F. (2006). Heat Stress-Induced H_2_O_2_ Is Required for Effective Expression of Heat Shock Genes in *Arabidopsis*. Plant Mol. Biol..

[22] Janicka M., Reda M., Czyżewska K., Kabała K. (2018). Involvement of Signalling Molecules NO, H_2_O_2_ and H_2_S in Modification of Plasma Membrane Proton Pump in Cucumber Roots Subjected to Salt or Low Temperature Stress. Funct. Plant Biol..

[23] Kabała K., Reda M., Wdowikowska A., Janicka M. (2022). Role of Plasma Membrane NADPH Oxidase in Response to Salt Stress in Cucumber Seedlings. Antioxidants.

[24] Velikova V., Yordanov I., Edreva A. (2000). Oxidative Stress and Some Antioxidant Systems in Acid Rain-Treated Bean Plants. Plant Sci..

[25] Kabała K., Zboińska M., Głowiak D., Reda M., Jakubowska D., Janicka M. (2019). Interaction between the Signaling Molecules Hydrogen Sulfide and Hydrogen Peroxide and Their Role in Vacuolar H^+^-ATPase Regulation in Cadmium-stressed Cucumber Roots. Physiol. Plant.

[26] Kabała K., Janicka-Russak M., Kłobus G. (2010). Different Responses of Tonoplast Proton Pumps in Cucumber Roots to Cadmium and Copper. J. Plant Physiol..

[27] Wdowikowska A., Reda M., Kabała K., Chohura P., Jurga A., Janiak K., Janicka M. (2023). Water and Nutrient Recovery for Cucumber Hydroponic Cultivation in Simultaneous Biological Treatment of Urine and Grey Water. Plants.

[28] Larsson C., Linskens H.F., Jackson J.F. (1985). Plasma Membranes. Cell Components.

[29] Kłobus G., Baluška F., Ciamporova M., Gasparicova O., Barlow P.W. (1995). The Role of Plasma Membrane-Bound Activities in Nitrate Transport into Sealed Plasma Membrane Vesicles from *Cucumis sativus* L. Roots. Developments in Plant and Soil Sciences.

[30] Gallagher S.R., Leonard R.T. (1982). Effect of Vanadate, Molybdate, and Azide on Membrane-Associated ATPase and Soluble Phosphatase Activities of Corn Roots. Plant Physiol..

[31] Kłobus G., Buczek J. (1995). The Role of Plasma Membrane Oxidoreductase Activity in Proton Transport. J. Plant Physiol..

[32] Janicka M., Reda M., Napieraj N., Michalak A., Jakubowska D., Kabała K. (2022). Involvement of Diamine Oxidase in Modification of Plasma Membrane Proton Pump Activity in *Cucumis sativus* L. Seedlings under Cadmium Stress. Int. J. Mol. Sci..

[33] Bradford M.M. (1976). A Rapid and Sensitive Method for the Quantitation of Microgram Quantities of Protein Utilizing the Principle of Protein-Dye Binding. Anal. Biochem..

[34] Li J., Chen G., Wang X., Zhang Y., Jia H., Bi Y. (2011). Glucose-6-Phosphate Dehydrogenase-Dependent Hydrogen Peroxide Production Is Involved in the Regulation of Plasma Membrane H^+^-ATPase and Na^+^/H^+^ Antiporter Protein in Salt-Stressed Callus from *Carex moorcroftii*. Physiol. Plant.

[35] Jakubowska D., Janicka-Russak M., Kabała K., Migocka M., Reda M. (2015). Modification of Plasma Membrane NADPH Oxidase Activity in Cucumber Seedling Roots in Response to Cadmium Stress. Plant Sci..

[36] Reda M. (2015). Response of Nitrate Reductase Activity and *NIA* Genes Expression in Roots of *Arabidopsis* Hxk1 Mutant Treated with Selected Carbon and Nitrogen Metabolites. Plant Sci..

[37] Kaiser W.M., Huber S.C. (1997). Correlation between Apparent Activation State of Nitrate Reductase (NR), NR Hysteresis and Degradation of NR Protein. J. Exp. Bot..

[38] Wdowikowska A., Klobus G. (2016). The Plasma Membrane Proton Pump Gene Family in Cucumber. Acta Physiol. Plant.

[39] Reda M., Migocka M., Kłobus G. (2011). Effect of Short-Term Salinity on the Nitrate Reductase Activity in Cucumber Roots. Plant Sci..

[40] Migocka M., Papierniak A. (2011). Identification of Suitable Reference Genes for Studying Gene Expression in Cucumber Plants Subjected to Abiotic Stress and Growth Regulators. Mol. Breed..

[41] Wasternack C., Hause B. (2013). Jasmonates: Biosynthesis, Perception, Signal Transduction and Action in Plant Stress Response, Growth and Development. An Update to the 2007 Review in Annals of Botany. Ann. Bot..

[42] Kim J., Chang C., Tucker M.L. (2015). To Grow Old: Regulatory Role of Ethylene and Jasmonic Acid in Senescence. Front. Plant Sci..

[43] Jang G., Chang S.H., Um T.Y., Lee S., Kim J.K., Choi Y.D. (2017). Antagonistic Interaction between Jasmonic Acid and Cytokinin in Xylem Development. Sci. Rep..

[44] Gasperini D., Chételat A., Acosta I.F., Goossens J., Pauwels L., Goossens A., Dreos R., Alfonso E., Farmer E.E. (2015). Multilayered Organization of Jasmonate Signalling in the Regulation of Root Growth. PLoS Genet..

[45] Ellouzi H., Hamed K.B., Cela J., Müller M., Abdelly C., Munné-Bosch S. (2013). Increased Sensitivity to Salt Stress in Tocopherol-Deficient *Arabidopsis* Mutants Growing in a Hydroponic System. Plant Signal Behav..

[46] Wasternack C., Strnad M. (2016). Jasmonate Signaling in Plant Stress Responses and Development–Active and Inactive Compounds. N. Biotechnol..

[47] Cai X.T., Xu P., Zhao P.X., Liu R., Yu L.H., Xiang C.B. (2014). *Arabidopsis* ERF109 Mediates Cross-Talk between Jasmonic Acid and Auxin Biosynthesis during Lateral Root Formation. Nat. Commun..

[48] Xu P., Zhao P.-X., Cai X.-T., Mao J.-L., Miao Z.-Q., Xiang C.-B. (2020). Integration of Jasmonic Acid and Ethylene into Auxin Signaling in Root Development. Front. Plant Sci..

[49] Yang D.-L., Yao J., Mei C.-S., Tong X.-H., Zeng L.-J., Li Q., Xiao L.-T., Sun T., Li J., Deng X.-W. (2012). Plant Hormone Jasmonate Prioritizes Defense over Growth by Interfering with Gibberellin Signaling Cascade. Proc. Natl. Acad. Sci. USA.

[50] Um T.Y., Lee H.Y., Lee S., Chang S.H., Chung P.J., Oh K.-B., Kim J.-K., Jang G., Choi Y.D. (2018). Jasmonate Zim-Domain Protein 9 Interacts with Slender Rice 1 to Mediate the Antagonistic Interaction Between Jasmonic and Gibberellic Acid Signals in Rice. Front. Plant Sci..

[51] Doke N. (1983). Involvement of Superoxide Anion Generation in the Hypersensitive Response of Potato Tuber Tissues to Infection with an Incompatible Race of *Phytophthora infestans* and to the Hyphal Wall Components. Physiol. Plant Pathol..

[52] Torres M.A., Jones J.D.G., Dangl J.L. (2006). Reactive Oxygen Species Signaling in Response to Pathogens. Plant Physiol..

[53] Hung K.T., Hsu Y.T., Kao C.H. (2006). Hydrogen Peroxide Is Involved in Methyl Jasmonate-Induced Senescence of Rice Leaves. Physiol. Plant.

[54] Sewelam N., Kazan K., Schenk P.M. (2016). Global Plant Stress Signaling: Reactive Oxygen Species at the Cross-Road. Front. Plant Sci..

[55] Jiang M., Xu F., Peng M., Huang F., Meng F. (2016). Methyl Jasmonate Regulated Diploid and Tetraploid Black Locust (*Robinia pseudoacacia* L.) Tolerance to Salt Stress. Acta Physiol. Plant.

[56] Faghih S., Ghobadi C., Zarei A. (2017). Response of Strawberry Plant Cv. ‘Camarosa’ to Salicylic Acid and Methyl Jasmonate Application Under Salt Stress Condition. J. Plant Growth Regul..

[57] Wu H., Wu X., Li Z., Duan L., Zhang M. (2012). Physiological Evaluation of Drought Stress Tolerance and Recovery in Cauliflower (*Brassica oleracea* L.) Seedlings Treated with Methyl Jasmonate and Coronatine. J. Plant Growth Regul..

[58] Abdelgawad Z.A., Khalafaallah A.A., Abdallah M.M. (2014). Impact of Methyl Jasmonate on Antioxidant Activity and Some Biochemical Aspects of Maize Plant Grown under Water Stress Condition. Agric. Sci..

[59] Yan Z., Zhang W., Chen J., Li X. (2015). Methyl Jasmonate Alleviates Cadmium Toxicity in *Solanum nigrum* by Regulating Metal Uptake and Antioxidative Capacity. Biol. Plant.

[60] Maruta T., Inoue T., Tamoi M., Yabuta Y., Yoshimura K., Ishikawa T., Shigeoka S. (2011). *Arabidopsis* NADPH Oxidases, AtrbohD and AtrbohF, Are Essential for Jasmonic Acid-Induced Expression of Genes Regulated by MYC2 Transcription Factor. Plant Sci..

[61] Ogawa K., Kanematsu S., Asada K. (1996). Intra-and Extra-Cellular Localization of “Cytosolic” CuZn-Superoxide Dismutase in Spinach Leaf and Hypocotyl. Plant Cell Physiol..

[62] Alscher R.G. (2002). Role of Superoxide Dismutases (SODs) in Controlling Oxidative Stress in Plants. J. Exp. Bot..

[63] Shapiguzov A., Vainonen J.P., Wrzaczek M., Kangasjärvi J. (2012). ROS-Talk–How the Apoplast, the Chloroplast, and the Nucleus Get the Message Through. Front. Plant Sci..

[64] Foyer C.H., Noctor G. (2011). Ascorbate and Glutathione: The Heart of the Redox Hub. Plant Physiol..

[65] Aguilar Diaz De Leon J., Borges C.R. (2020). Evaluation of Oxidative Stress in Biological Samples Using the Thiobarbituric Acid Reactive Substances Assay. J. Vis. Exp..

[66] Sirhindi G., Mir M.A., Sharma P., Gill S.S., Kaur H., Mushtaq R. (2015). Modulatory Role of Jasmonic Acid on Photosynthetic Pigments, Antioxidants and Stress Markers of *Glycine max* L. under Nickel Stress. Physiol. Mol. Biol. Plants.

[67] Scharte J., Schön H., Tjaden Z., Weis E., von Schaewen A. (2009). Isoenzyme Replacement of Glucose-6-Phosphate Dehydrogenase in the Cytosol Improves Stress Tolerance in Plants. Proc. Natl. Acad. Sci. USA.

[68] Liu S., Cheng Y., Zhang X., Guan Q., Nishiuchi S., Hase K., Takano T. (2007). Expression of an NADP-Malic Enzyme Gene in Rice (*Oryza sativa* L.) Is Induced by Environmental Stresses; over-Expression of the Gene in *Arabidopsis* Confers Salt and Osmotic Stress Tolerance. Plant Mol. Biol..

[69] Zhu C., Yang N., Ma X., Li G., Qian M., Ng D., Xia K., Gan L. (2015). Plasma Membrane H^+^-ATPase Is Involved in Methyl Jasmonate-Induced Root Hair Formation in Lettuce (*Lactuca sativa* L.) Seedlings. Plant Cell Rep..

[70] Schaller A., Oecking C. (1999). Modulation of Plasma Membrane H^+^-ATPase Activity Differentially Activates Wound and Pathogen Defense Responses in Tomato Plants. Plant Cell.

[71] Janicka-Russak M., Kabała K., Wdowikowska A., Kłobus G. (2012). Response of Plasma Membrane H^+^-ATPase to Low Temperature in Cucumber Roots. J. Plant Res..

[72] Zboińska M., Romero L.C., Gotor C., Kabała K. (2023). Regulation of V-ATPase by Jasmonic Acid: Possible Role of Persulfidation. Int. J. Mol. Sci..

[73] Wu X., Ding C., Baerson S.R., Lian F., Lin X., Zhang L., Wu C., Hwang S.-Y., Zeng R., Song Y. (2019). The Roles of Jasmonate Signalling in Nitrogen Uptake and Allocation in Rice (*Oryza sativa* L.). Plant Cell Environ..

[74] Orsel M., Chopin F., Leleu O., Smith S.J., Krapp A., Daniel-Vedele F., Miller A.J. (2006). Characterization of a Two-Component High-Affinity Nitrate Uptake System in *Arabidopsis*. Physiology and Protein-Protein Interaction. Plant Physiol..

[75] Lupini A., Mercati F., Araniti F., Miller A.J., Sunseri F., Abenavoli M.R. (2016). NAR2.1/NRT2.1 Functional Interaction with NO_3_^−^ and H^+^ Fluxes in High-Affinity Nitrate Transport in Maize Root Regions. Plant Physiol. Biochem..

[76] Orsel M., Krapp A., Daniel-Vedele F. (2002). Analysis of the NRT2 Nitrate Transporter Family in *Arabidopsis*. Structure and Gene Expression. Plant Physiol..

[77] Campbell W.H. (1999). Nitrate Reductase Structure, Function and Regulation: Bridging the Gap between Biochemistry and Physiology. Annu. Rev. Plant Physiol. Plant Mol. Biol..

[78] Yu X., Fei P., Xie Z., Zhang W., Zhao Q., Zhang X. (2019). Effects of Methyl Jasmonate on Growth, Antioxidants, and Carbon and Nitrogen Metabolism of *Glycyrrhiza uralensis* under Salt Stress. Biol. Plant.

[79] Farhangi-Abriz S., Ghassemi-Golezani K. (2016). Improving Amino Acid Composition of Soybean under Salt Stress by Salicylic Acid and Jasmonic Acid. J. Appl. Bot. Food Qual..

