# Supplementary material for: Jasmonic Acid Effect on Cucumis sativus L. Growth Is Related to Inhibition of Plasma Membrane Proton Pump and the Uptake and Assimilation of Nitrates
Source: Cells. 2023 Sep 13;12(18):2263. doi: 10.3390/cells12182263 (PMC10526807; doi:10.3390/cells12182263)
Supplement: Supplementary file 1 [file cells-12-02263-s001.zip › cells-2575749-supplementary.pdf]

Supplementary file

Jasmonic acid effect on *Cucumis sativus* L. growth is related to inhibition of plasma membrane proton pump and the uptake and assimilation of nitrates

Małgorzata Janicka [1], Małgorzata Reda [1], Emilia Mroczko [1], Anna Wdowikowska[1] and Katarzyna Kabała [1,*]

[1] Department of Plant Molecular Physiology, Faculty of Biological Sciences, University of Wrocław, Kanonia 6/8, 50-328 Wrocław, Poland

* Correspondence: katarzyna.kabala@uwr.edu.pl

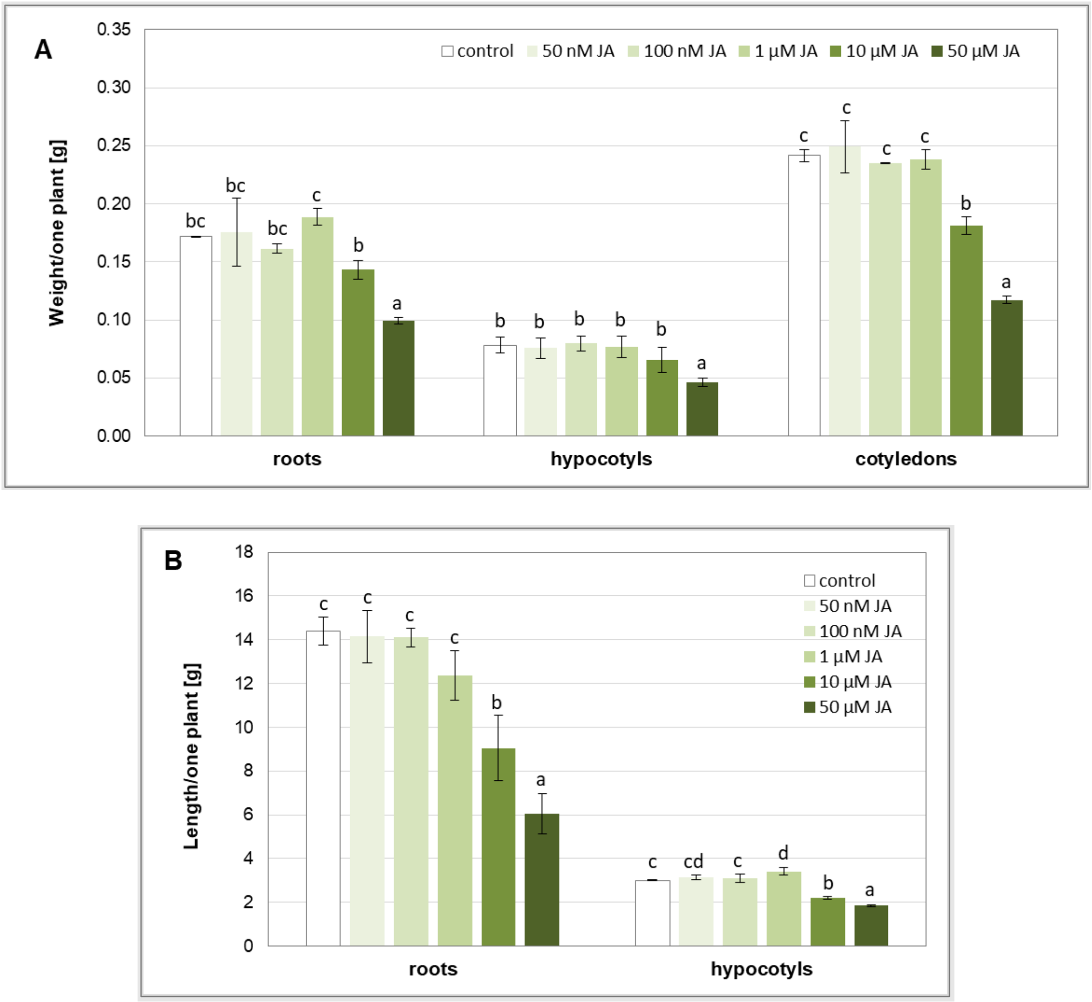

Figure S1. Weight (A) and length (B) of individual organs of cucumber seedlings exposed to jasmonic acid (JA) for 6 d. Plants were treated with 50 nM, 100 nM, 1 µM, 10 µM and 50 µM JA or without this hormone (control). Data represent the means of 4 biological repetitions (each containing 10 plants) ± SD. Different letters represent homogeneous groups, independently for each organ, according to Duncan's test ($p<0.05$).

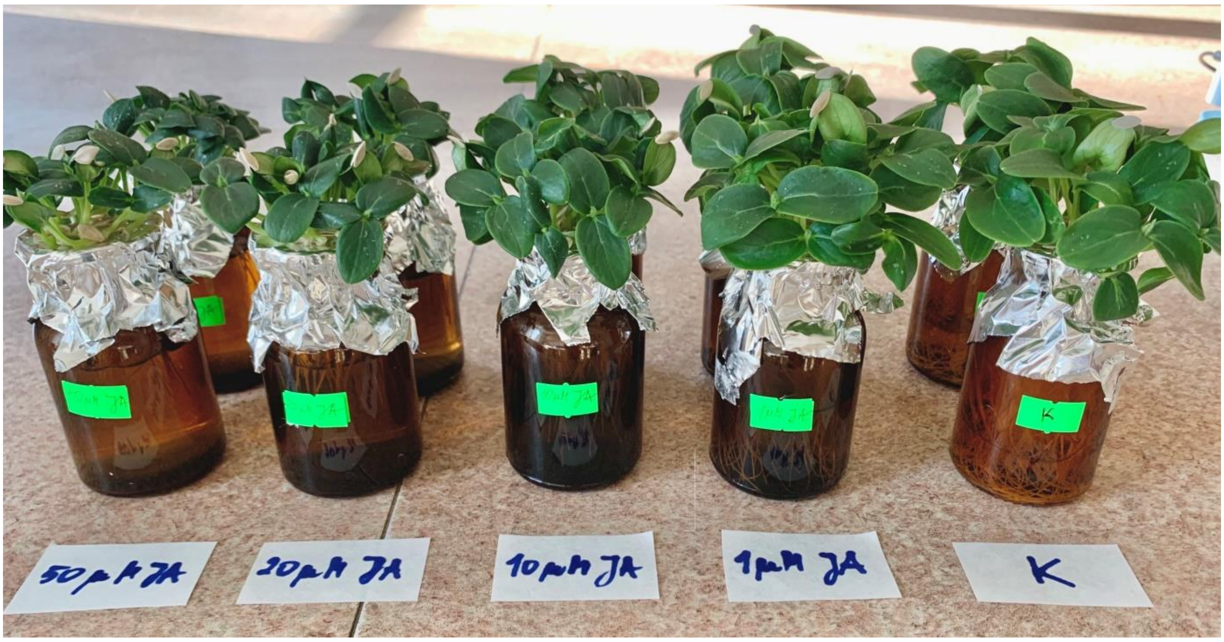

Figure S2. Representative image of cucumber seedlings treated with different concentrations of jasmonc acid (JA) for 6 d.

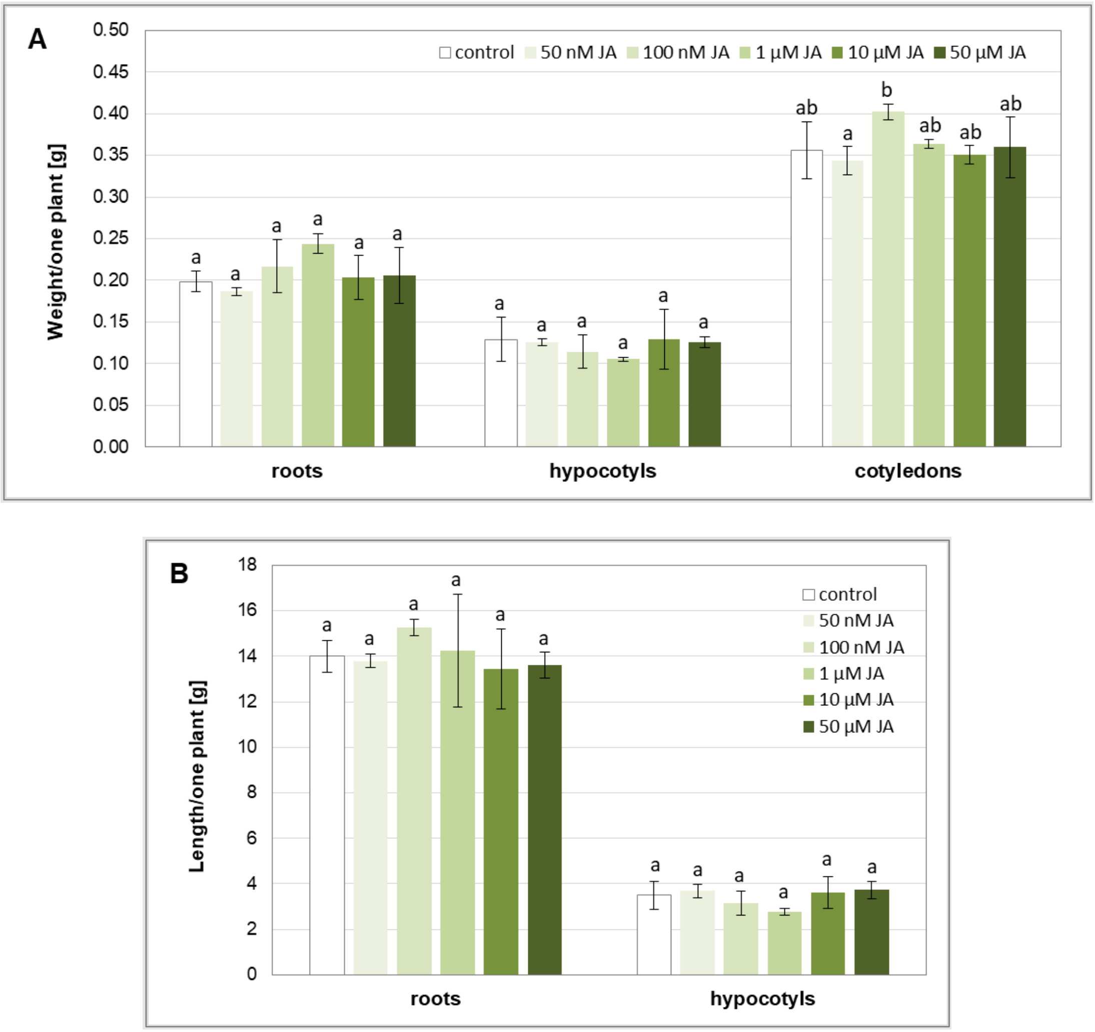

Figure S3. Weight (A) and length (B) of individual organs of cucumber seedlings exposed to jasmonic acid (JA) for 24 h. Plants were treated with 50 nM, 100 nM, 1 µM, 10 µM and 50 µM JA or without this hormone (control). Data represent the means of 2 biological repetitions (each containing 10 plants) ± SD. Different letters represent homogeneous groups, independently for each organ, according to Duncan's test ($p<0.05$).

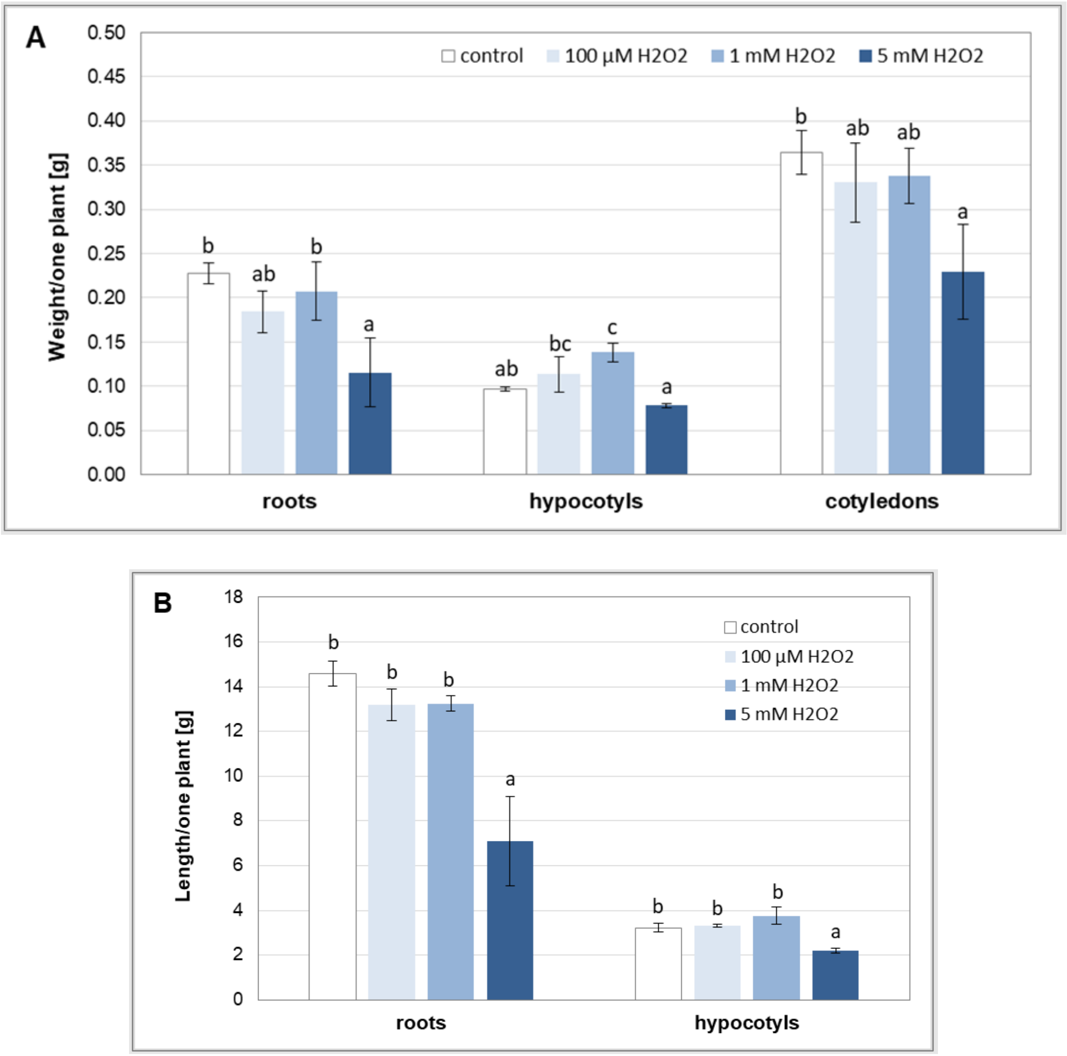

Figure S4. Weight (A) and length (B) of individual organs of cucumber seedlings exposed to H_2O_2 for 6 d. Plants were treated with 100 µM, 1 mM and 5 mM H_2O_2 or without this compound (control). Data represent the means of 2 biological repetitions (each containing 10 plants) ± SD. Different letters represent homogeneous, independently for each organ, groups according to Duncan's test ($p<0.05$).

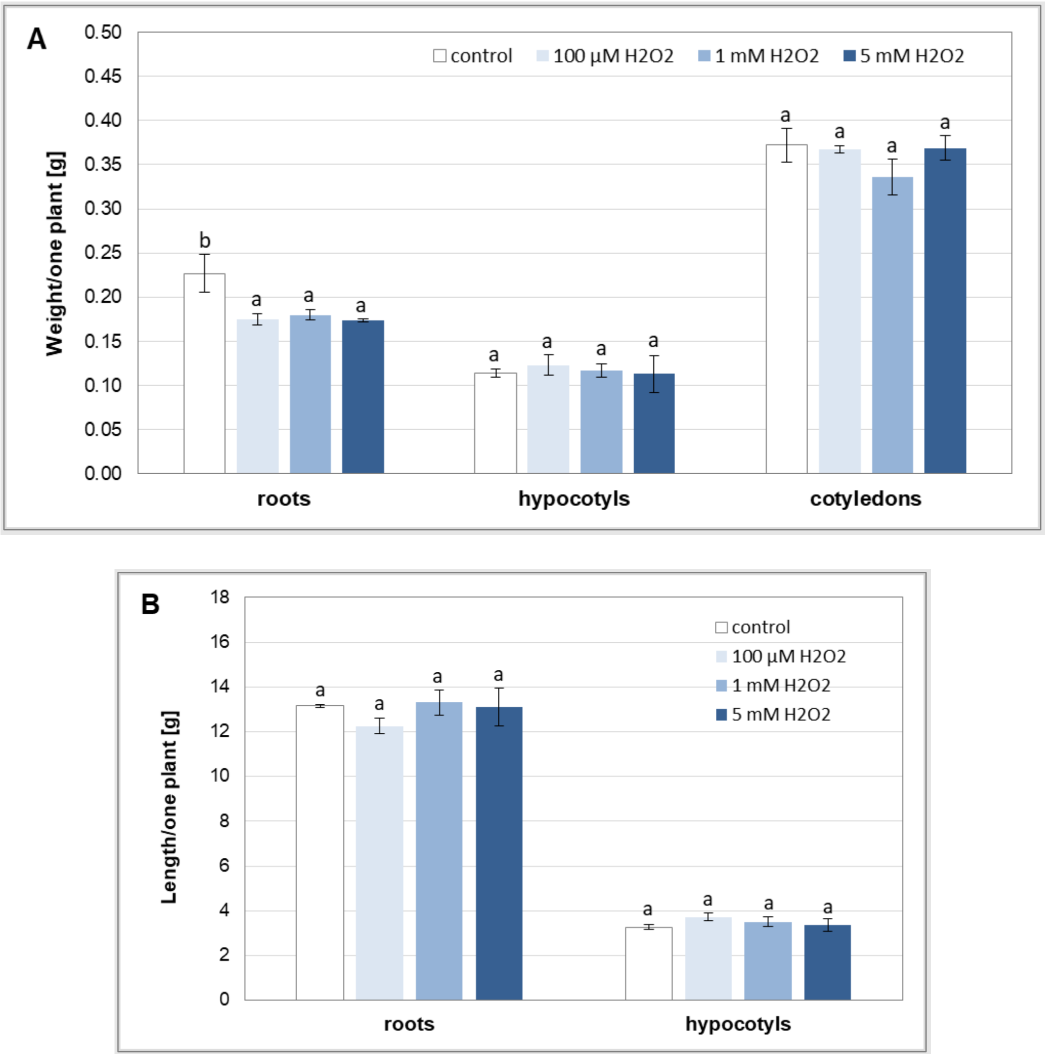

Figure S5. Weight (A) and length (B) of individual organs of cucumber seedlings exposed to H_2O_2 for 24 h. Plants were treated with 100 µM, 1 mM and 5 mM H_2O_2 or without this compound (control). Data represent the means of 2 biological repetitions (each containing 10 plants) ± SD. Different letters represent homogeneous groups, independently for each organ, according to Duncan's test ($p<0.05$).

Table S1. The primers used in qRT-PCR analysis.

Gene	Accession No.	Forward primer (5′ – 3′)	Reverse primer (5′ – 3′)	Temp. of annealing	References
CsHA1	JK693835	CGTGTTCCGTTCACTCACTT	GCATTTGAGTTTCTCAAACACATCAT	58 °C	[38]
CsHA2	EU735752	ACCCGAGTCGACAAACATCT	CTTGGCACAGCAAAGTGAAA		
CsHA3	EF375892	AAGTTTCTGGGGTTCATGTGGAAT	GTAACAGGAAGTGACTCTCCAGTC		
CsHA4	HO054960	CTACAGCTTGGTAACATACATTC	GTTGTAGTCCATGTAATGTCCTC		
CsHA8	HO054964	CTCATGCGCAAAGAACATTAC	CTGAATTGTGTCAATGTCAAGTC		
CsHA9	HO054965	AAACCAGAAGTGCTGGAG	CTCAGCACCCTCACTAGTAA		
CsHA10	HO054966	GACATAATCAAGTTTGCAATCAGATA	TTCTGTATAAGTTGTGCGGT		
CsRBOHB	JZ763593	AGAGTCGGCTTCAGCGG	TCTCCTCCTCTGATGTTCTGAACGG	57 °C	[35]
CsRBOHD	JZ763594	TCTTCTTCTTCTTCCTCCCTCAAAGCC	GAAAGTTCAGGGTCTTCAAGAGAGTTG		
CsRBOHE	JZ763595	TCACTTTGGAAGTTGAGGATGATTCTGTT	GTCGAGAGAGGAGGCAGTGG		
CsRBOHF1	JZ763596	GCTTCGATTTCGAGATCGCCGAC	AAATCCATTTCCACCACCAGAAGAATGT		
CsRBOHF2	JZ763597	TCGCATCGGTTTCGGCAG	AGTTCACGGTTGAAGATGACGC		
CsRBOHF3	JZ763598	AAGAGAAGTCCGAGAATGAGGTTTAT	CTCCTGAGATTCTGACATTCCAATGC		
CsRBOHH1	JZ763599	AGATTCCGATGTGATAGATGTCATGGT	TTGGACTGTCTTCTTCCAAGCCTC		
CsRBOHH2	JZ763600	AAATCCAGGAGAATGCCCAC	TGAGAAGTCTCAGCGACGG		
CsRBOHJ	JZ763601	CTGACGATGGGATTACTCTGCAACA	GAAGCAGTCCACTTCAGTATGGTCT		
CsCSD1	ACYN01000940.1 (2566-4619 nn)	AAAGATGGTGAAGGCTGTGG	CATGTTGTTTTCCAGCAG	58 °C	[23]
CsCSD2	ACYN01004691.1 (4929-8291 nn)	CCCATTCTCCAATTCTTCAT	TTGGGTGAGCGTGACAA		
CsCSD3	ACYN01007278.1 (4575-9323 nn)	CCAAAAGAGGGGGAATTTTT	GCCTCTGACGTTGGAATCTC		
CsMSD	ACYN01004451.1 (16823-20273 nn)	CGCTTCGAATTCTAGGCAGG	CCTTTGTTGATGGCCTCGTG		
CsFSD2	ACYN01004137.1 (18052-22184 nn)	TAACACGGTTGACCATCC	TTCTGTAGCCTCAAGTCT		
CsFSD3	ACYN01000936.1 (63338-67558 nn)	TCTGATGGAACCACAAGAG	TGTGGCCATATAGAATATCATTT		
CsNR1	HM755943	GGACGGTAGAGTAAAGAAGGC	TATCCCTTTTACTCCATTCA	56 °C	[39]
CsNR2	HM755944	CGACTCCTCCTCCAACTCC	CCACTTCCATGTTGTCCAA		
CsNR3	JQ692875	TCCAATGGCGACTGCTG	CATCATCATCAATAAGGAGCGG		
CsNRT2.1	AY584189	CTCATAATACAACTAAAGCCAAAAC	TGTAACCAGCAGCATTCGAC	62 °C	-
CsNRT2.2	KC783255	TCGTTCATTCTTCAGCCAAAACAACA	TGTAACCAGCAGCATTCG		
CsNRT2.3	KC783256	CGCCATAATCGAACACATTG	GCAATAGAAGAACTCAAAGAATCAACCAATC		
CsNAR2	GQ402328	CTTTGGAGGTCACTGCTTC	CGTCTTGTTCGCTGGATTGTA		
CsCACS	GW881874	TGGGAAGATTCTTATGAAGTGC	CTCGTCAAATTTACACATTGGT	56-62 °C	[40]

Table S2. Transcript level of *CsRBOH* genes in roots of cucumber control plants.

Gene	Transcript level ± SD
CSRBOHB	2.725 ± 0.859
CSRBOHD	2.424 ± 0.446
CSRBOHE	0.0888 ± 0.0011
CSRBOHF1	0.277 ± 0.043
CSRBOHF2	0.261 ± 0.036
CSRBOHF3	0.332 ± 0.065
CSRBOHH1	0.0014 ± 0.0003
CSRBOHH2	0.323 ± 0.030
CSRBOHJ	0.0856 ± 0.0128

The results are means ± SD of 6 replications of 2 independent experiments.

Table S3. Transcript level of genes encoding SOD isoforms in roots of cucumber control plants.

Gene	Transcript level ± SD
CsCSD1	3.250 ± 0.413
CsCSD2	0.436 ± 0.024
CsCSD3	0.004 ± 0.001
CsMSD	1.280 ± 0.150
CSFSD2	0.112 ± 0.014
CsFSD3	0.099 ± 0.008

The *CsCSD1-3* encode Cu-ZnSOD isoforms; *CsMSD* encodes Mn-SOD isoform; *CsFe2-3* encode Fe-SOD isoforms. The results are means ± SD of 6 replications of 2 independent experiments.

Table S4. Transcript level of *CsHA* genes in roots of cucumber control plants.

Gene	Transcript level ± SD
CsHA1	0.0017 ± 0.0003
CsHA2	9.387 ± 2.123
CsHA3	6.617 ± 1.169
CsHA4	0.059 ± 0.015
CsHA8	2.502 ± 0.407
CsHA9	1.122 ± 0.139
CsHA10	0.073 ± 0.034

The results are means ± SD of 4-6 replications of 2 independent experiments.

Table S5. Transcript level of *CsNR* genes in roots of cucumber control plants.

Gene	Transcript level ± SD
CsNR1	0.301 ± 0.127
CsNR2	1.516 ± 0.361
CsNR3	0.243 ± 0.078

The results are means ± SD of 6-7 replications of 2 independent experiments.
